# To err is human… to forgive and remember is divine?

**DOI:** 10.4103/0019-5049.76574

**Published:** 2011

**Authors:** S Bala Bhaskar

**Affiliations:** Department of Anaesthesiology & Critical Care, Vijayanagar Institute of Medical Sciences (VIMS), Bellary, Karnataka, India

Sir,

The Indian Journal of Anaesthesia should be congratulated for publishing the Guest Editorial by Frederick van Pelt on a subject not normally discussed.[1] The background leading to the launch of his noble initiative, Medically Induced Trauma Support Services (MITSS), with active participation from the patient Ms. Linda Kenney, who survived physical and psychological insults, along with her family members, due to medical error makes me wonder if this is real. That there was concealment of the facts of the case by the administrators, and secondly, an overall complicity by professional colleagues in a western hospital is surprising. This does not, however, take away the excellent concept put into our minds by the article. In the Indian scenario, there is indeed a tendency both at a personal level and at an institutional level to deprive the patient or the attendants, the full facts of a mishap resulting from medical error. Such a “shared responsibility” by the medical service providers may be self-benefiting in the short term but as the author says, it takes a lot to “unburden” this emotional load at a personal level. The stress would be amplified greatly by having to work within the constraints of limited resources at different levels of health care. It was heartening to see sections devoted to clinicians and health care organisations in the website of MITSS launched by Frederick van Pelt.[2] The admission of error is a noble principle; but I am afraid, in India, ignorance and illiteracy may lead to more stress and even endanger the care giver. The care givers are likely to desist from well-intentioned explanations and admissions, let alone apologise, because of lack of proper and timely implementation of the laws of the land by the authorities. I feel we are still far away from launching similar programmes. To err is human, to forgive is divine (Alexander Pope: 1688–1744) is more apt in letter than in practice; however, bringing down the rate of error should be the first target among health care professionals across the world. “To err is human: Building a safer health system”[3] is an excellent report issued by the Institute of Medicine (USA) intended to promote increased awareness of US medical errors. To quote “Errors … are costly in terms of loss of trust in the health care system by patients and diminished satisfaction by both patients and health professionals”.[3] The follow-up reports of US Institute of Medicine may explain what the future holds for such endeavors to reduce medical errors.[3,4]
